# The Coexistence of Genetic Mutations in Thyroid Carcinoma Predicts Histopathological Factors Associated With a Poor Prognosis: A Systematic Review and Network Meta-Analysis

**DOI:** 10.3389/fonc.2020.540238

**Published:** 2020-11-03

**Authors:** Ling Zhao, Lin Wang, Xiaomeng Jia, Xiaodong Hu, Ping Pang, Sitong Zhao, Yajing Wang, Jing Wang, Yingshi Zhang, Zhaohui Lyu

**Affiliations:** ^1^ The Department and Key Laboratory of Endocrinology and Metabolism, The First Medical Center of PLA General Hospital, Beijing, China; ^2^ Department of Endocrinology, The 940th Hospital of Joint Logistics Support force of Chinese PLA, Lanzhou, China; ^3^ Department of Endocrinology, Hainan Hospital of PLA General Hospital, Sanya, China; ^4^ The 8th Medical Center of Chinese PLA General Hospital, Beijing, China; ^5^ Department of Clinical Pharmacy, Shenyang Pharmaceutical University, Shenyang, China

**Keywords:** coexistent genetic mutations, thyroid carcinoma, histopathological features, prognosis, *BRAF^V600E^* + *TERT*

## Abstract

**Purpose:**

Genetic mutations may play an important role in the progression and invasion of thyroid carcinoma (TC), and their coexistence may result in mutational synergy. The presence of the *BRAF^V600E^* mutation, as well as mutations affecting the *TERT* promoter, *RAS*, *CHEK2* and *RET/PTC*, may all have an impact on prognosis. The aim of this study was to explore whether synergy between the coexistent mutations predicts histopathological prognostic factors that influence disease outcome.

**Methods:**

A comprehensive literature search of PubMed, Embase and the Cochrane Library, from their inception until January 2020. Primary outcomes included: disease stage, lymph node metastasis, extrathyroidal extension and distant metastasis; while, secondary outcomes included: tumor recurrence, mortality, invasion of thyroid capsule, multiplicity, presented as an odds ratio (OR) with 95% credible intervals (CrI).

**Results:**

27 publications (comprising 9 active intervention arms), involving 8,388 TC patients, were selected. Network meta-analytic estimates of active interventions contrasted with other active interventions, with random effects, were calculated. In terms of outcomes focus on overall TC, *BRAF^V600E^* + *TERT* co-mutation ranked highest for diseases stage (OR = 5.74, 95% CrI: 3.09–10.66), as well as lymph node metastasis, extrathyroidal extension (5.74, 4.06–8.10), tumor recurrence (7.21, 3.59–14.47), and invasion of the thyroid capsule (3.11, 1.95–4.95). *BRAF^V600E^* + *TERT* co-mutation ranked secondary in distant metastasis, mortality, and multiplicity that ranked highest was *TERT*+*RAS* or *RAS*. When we were limited to the study of patients with papillary TC (PTC), *BRAF^V600E^* + TERT always ranked highest for primary outcomes: disease stage (6.39, 3.13–13.04), lymph node metastasis, extrathyroidal extension (5.80,3.89–8.64) and distant metastasis (7.33, 3.00–17.89), while *BRAF^V600E^* + *TERT* again ranked highest in secondary outcomes: tumor recurrence (7.23,3.37–15.51), mortality (9.26, 3.02–28.42), invasion of thyroid capsule (3.20,2.01–5.11), and multiplicity.

**Conclusions:**

In this molecular marker mutation-based systematic review and network meta-analysis, we found that coexistent *BRAF^V600E^* + *TERT* genetic co-mutations predicted poor histopathological prognosis, including progression, invasion, and metastasis, especially in PTC. For the overall TC, the *BRAF^V600E^* + *TERT* + *RAS* triple mutations may have a greater impact on the prognosis, and further research should related to potentially important features. This study is registered with PROSPERO, number CRD42019143242.

## Introduction

Thyroid carcinoma (TC) is the most common type of endocrine malignancy, the incidence of which, has undergone a steady increase over the last two decades worldwide, becoming the sixth leading cause of malignant neoplasms in women (1). According to the various molecular origins of TC, its pathological type and sub-type can be defined as either papillary thyroid carcinoma (PTC), follicular thyroid carcinoma (FTC), poorly differentiated thyroid carcinoma (PDTC), or anaplastic thyroid carcinoma (ATC). In addition, medullary thyroid carcinoma (MTC), which originates from parafollicular cells, also accounts for a small proportion of thyroid malignancies. Differentiated thyroid cancer mainly includes PTC and FTC, of which, PTC represents the most common clinical pathological type, accounting for more than 80% of all TC cases (2, 3). Although at present, the mortality rate for TC has not risen rapidly, as the degree of malignancy is generally low, meaning that the majority of TC patients achieve a good therapeutic outcome. For patients with no metastasis, surgery represents in usually the first-line treatment. Although the differentiation of TC is good, the degree of malignancy is low, and I^131^ treatment is the main treatment after traditional thyroidectomy or near-total thyroidectomy. However, some TC (especially PTC) tumors are highly invasive, postoperative recurrence, metastasis or even death occur frequently. Therefore, novel therapeutic strategies are urgently needed (4, 5).

In the era of precision medicine, the ultimate goal pursued by clinicians is to accurately assess the patient’s condition and prepare the most appropriate individualized treatment plan (6). Therefore, research on the mutational profile in thyroid carcinomas is a priority. Recent medical research has resulted in great progress in the study of thyroid tumorigenesis at the molecular level. Numerous studies have found that certain genetic mutations are significantly correlated with the development, progression, prognosis, and diagnosis of TC (7–10). Moreover, the coexistence of several key mutations may lead to mutational synergy. Therefore, there is an increasing requirement for more accurate prognostic molecular markers, to be used as tools in the prediction of histopathological prognostic factors, which may impact disease outcome.

Genetic mutations play an important role in the etiology, progression, and invasion of TC. To this end, recent studies have focused on the identification of genetic mutations as molecular markers, which will of utmost clinical importance in predicting the progression and prognosis of TC (7–10). Mutations targeting components of the well-characterized mitogen-activated protein kinase (MAPK) signaling pathway have been identified as driver mutations (11, 12). Molecular alterations affecting MAPK signaling include: i) point mutations in the B-Raf proto-oncogene (*BRAF*) and *RAS* genes, ii) chromosomal rearrangements of *RET*/papillary thyroid cancer (PTC) and PAX8/peroxisome proliferator-activated receptor γ (PPARγ) (13, 14), and iii) the recently identified Telomerase Reverse Transcriptase (*TERT*) promoter mutations (15, 16). The most frequently-occurring mutation in the *BRAF* gene is V600E (*BRAF* ^V600E^), which promotes the constitutive activation of *BRAF* kinase (17, 18) and is widely accepted as a highly specific molecular marker for PTC.

Although several studies have shown that these individual genetic mutations may be associated with certain histopathological features and outcomes (19, 20), their coexistence may have a synergistic effect, thus having a higher impact on disease prognosis. Moon et al. demonstrated that coexistence of the *BRAF* ^V600E^ and *TERT* promoter mutations has a synergistic effect on the clinical outcomes in PTC, whereas each mutation alone exerts only a modest effect (21). Therefore, the aim of our systematic review and network meta-analysis was to provide a more accurate measure of TC prognosis by identifying the impact of coexisting mutations.

## Methods

This network meta-analysis followed the Preferred Reporting Items for Systematic Reviews and Meta-Analyses (PRISMA) guidelines and PRISMA extension guidelines (22, 23). A prospective protocol was created and uploaded to the PROSPERO online platform using the registration number CRD42019143242 (24).

### Search Strategy

To perform the systematic review and network meta-analysis, we searched PubMed, Embase and the Cochrane Library for relevant records published in English and Chinese (from database inception date to January 2020) using the search terms “genetic mutations” OR “gene mutations” AND “thyroid carcinoma” OR “thyroid cancer,” and their Medical Subject Headings (MeSH) terms combined with a list of all included studies (see details in **Supplementary Table 1**). We included clinical data comparing coexistent genetic mutations with single genetic mutations as molecular markers for predicting the histopathological features associated with prognosis.

### Eligibility and Exclusion Criteria

Studies had to include at least two of the following genetic mutations molecular marker types: the *BRAF^V600E^* gene mutation, *TERT* promoter mutations, *RAS* gene mutations, *CHEK2* mutations and *RET/PTC* gene rearrangements. Participants had to be adults (≥18 years old and of both genders) with a primary diagnosis of TC, with no specific TC type restrictions. We excluded conference abstracts, reviews, meta-analyses, letters, and records, which did not meet our criteria, such as not reporting coexisting genetic mutations etc. After removing duplicate records and performing a preliminary screening of titles and abstracts, two researchers (WL and ZL) independently assessed full-text and supplementary materials of the selected records for final inclusion. Potentially relevant full-text published articles were also retrieved and assessed. Disagreements were resolved by consensus or by requesting an additional round of reviewing by ZYS or LZH.

## Data Extraction

Although the ‘histopathological features associated with a worsened prognosis’ was our outcome of interest, this term was too broad for describing mutation-specific TC phenotypes. Instead, we broke the term ‘histopathological features associated with worsened prognosis’ into primary outcomes, such as disease stage, lymph node metastasis, extrathyroidal extension, and distant metastasis, and secondary outcomes including tumor recurrence, mortality, invasion of the thyroid capsule, and multiplicity. These outcomes were deemed to represent a suitable alternative for assessing histopathological prognostic features in the majority of the selected studies.

Two researchers (LW and LZ) independently used a standardized electronic form to extract and summarize the following data: study first author, publication year, region, TC type, sample size, specimen type, detection method, molecular markers (coexisting mutations, single mutations, and no mutation), and available outcomes.

## Quality Assessment and the Grading of Recommendations Assessment, Development and Evaluation (GRADE) Rating Scale

Two reviewers (LW and LZ) evaluated the risk of bias in our analyses, based on the original records and their supplementary materials, using the Critical Appraisal Skills Programme (CASP) scales (25), which were designed for the assessment of observational studies. Twelve aspects were assigned an assessment index associated with the risk of bias as ‘yes,’ ‘no,’ or ‘cannot tell.’ Moreover, we used the GRADE framework to develop and present summaries of evidence (26).

## Data Synthesis and Statistical Analysis

To estimate the effect sizes for the categorical outcomes using our outcome data, we computed the odds ratio (OR) with 95% confidence intervals (CI, for standardized meta-analysis) and 95% credible intervals (CrI, for network meta-analysis). In order to address heterogeneity relating to the outcomes documented in each of the selected study records, we used the random effects model, which is best suited to resolving heterogeneity in standardized meta-analyses (27, 28), to record the two-sided *P* value and *I* ^2^ statistic (the ratio of true heterogeneity to total observed variation) measures.

To visualize network geometry and node connectivity, we generated network plots for the primary outcomes. Moreover, we undertook consistency testing *via* both direct and indirect evidence using the random effects model, and were satisfied with the level of consistency in our network meta-analysis. We use the inconsistency factor (IF) to determine the factors that affect the authenticity of network meta-analysis. If the IF value is close to 0, then it means that direct evidence and indirect evidence are very consistent. Mean rank and surface under the cumulative ranking curve (SUCRA) values were produced for primary and secondary outcomes (29, 30). Publication bias was determined by adjusting funnel plot asymmetry. Meta-analysis was carried out using the “mvmeta” and “network” packages of Stata MP software, version 14.0.

## Results

### Systematic Review and Characteristics

**Figure 1:**
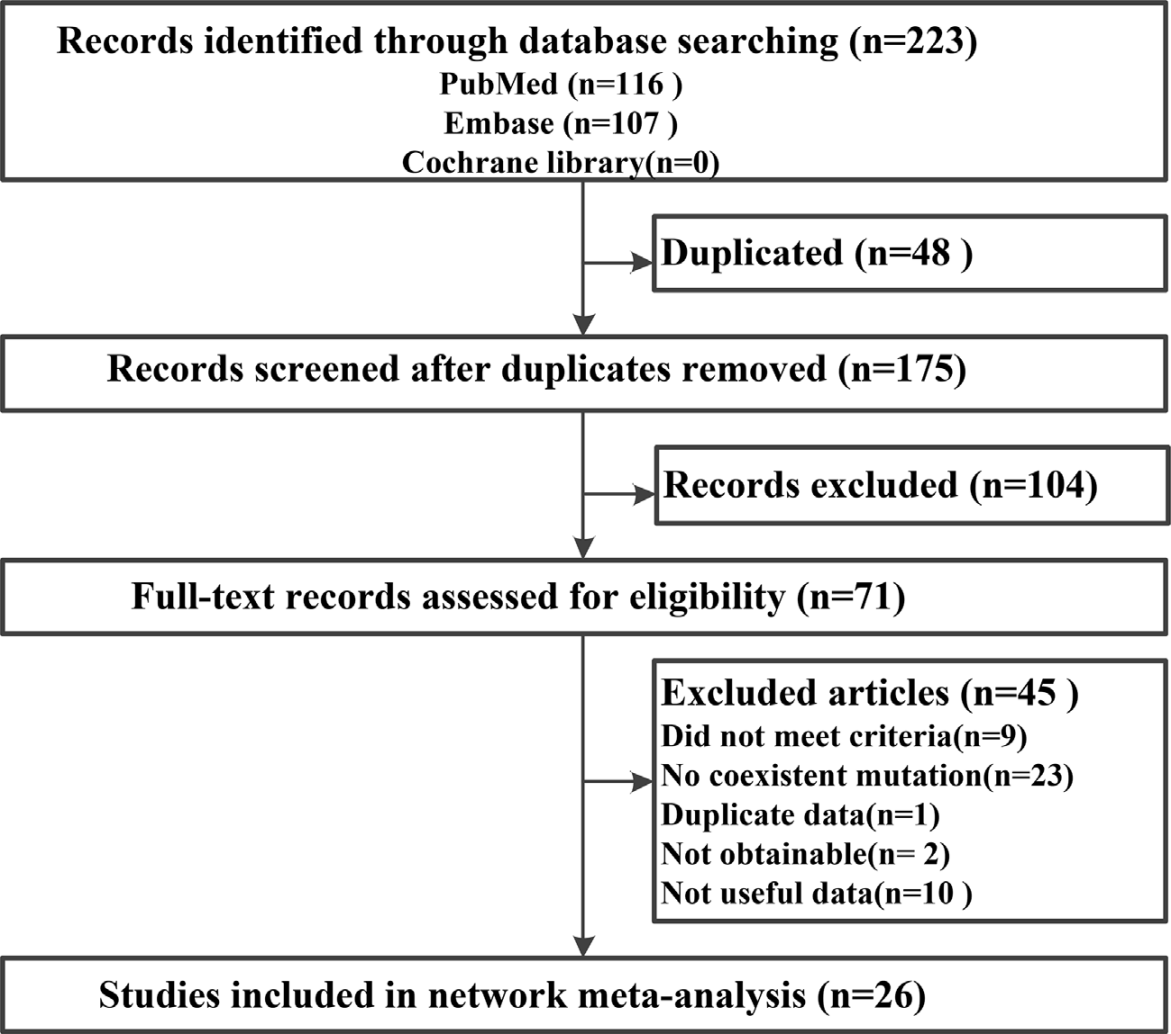
A flowchart summarizing all study assessment processes.

Electronic searches identified a total of 223 potentially eligible records. Following the elimination of duplicate records and a preliminary review, 71 full-text records were assessed. Further exclusion of unsuitable articles yielded a final 26 studies (31–56) for use in network meta-analysis (**Figure 1**). Overall, data relating to the histopathological features collected from 8,388 patients and documented in 26 studies met our inclusion criteria (**Table 1**).

**Table 1:** Summary active comparisons for coexistent.

Study, Year	Region	Thyroid cancer type	Sample size(male/female)	Age	Specimen(FNAB,Ppostsurgical)	Detection Method	Molecular markers										Available Outcomes							
							coexistent mutations				Single mutation					No mutation	Disease stage	Lymph node metastasis	Extrathyoidal extension	Distant metastasis	Tumor recurrence	Mortality	Invasion of the thyroid capsule	Multiplicity
							*BRAF^V600E^+TERT*	*TERT+RAS*	*BRAF^V600E^+RET/RTC*	*BRAF^V600E^+CHEK2*	*BRAF^V600E^*	*TERT*	*RAS*	*RET/RTC*	*CHEK2*	Wild-type								
Colombo et al. (31)	Italy	PTC	208(51/157)	–	Postsurigical	DNA sequencing	√				√	√				√		√	√					√
Gąsior-Perczak et al. (32)	Poland	PTC	427(50/377)	48.5 ± 12.3	Postsurigical	PCR				√	√				√	√	√	√	√	√	√		√	√
Giorgenon et al. (33)	Brasil	PTC	45(39/6)	–	FNAB	DNA sequencing	√				√					√	√	√	√				√	√
Hou et al. (34)	China	PTC	220(70/150)	45.69 ± 12.92	Postsurigical	PCR	√				√	√				√		√	√				√	
Huang et al. (35)	China	PTC	483(127/356)	43.15 ± 11.25	Postsurigical	PCR	√				√					√		√						√
Song et al. (36)	China	PTC	264(67/197) 64(15/49)	–		TCGA	√				√					√	√	√	√	√	√	√		√
Argyropoulou et al. (37)	Greece,	PTC	59	–	Postsurigical	PCR and DNA sequencing	√				√	√	√			√	√	√	√					
Dai (38)	China	PTC	62(18/44)	7–79	Postsurigical	PCR	√				√	√				√		√	√				√	
Deng (39)	China	PTC	432(113/319)		Postsurigical	PCR	√				√	√						√						
Ren (40)	China	PTC	342(99/243)	42.4 ± 13.2	FNAB and postsurgical	DNA sequencing	√				√	√				√	√	√	√					√
Rusinek et al. (41)	Poland	PTC	189(23/166)	42.4 ± 13.2	FNAB and postsurgical	DNA sequencing	√				√	√				√	√	√					√	√
Zhou et al. (42)	China	PTC	50(14/36)	53.06 ± 11.42	FNAB	PCR			√		√			√				√						
Liu et al. (43)	USA	PTC	1051(764/287)	46 (36–57)	Postsurigical	PCR and DNA sequencing	√				√	√				√						√		
Marques et al. (44)	Portugal	FNMTC	54(9/45)	–	–	PCR and DNA sequencing	√				√		√			√	√	√	√	√	√	√	√	√
Shen et al. (45)	USA,multi-centre	PTC	388(89/299)	46–66	FNAB and postsurgical	DNA sequencing	√	√			√	√	√			√	√	√	√	√	√	√		√
Song et al. (46)	Korea	FTC	690(56/634)	>40	FNAB	DNA sequencing		√				√	√			√					√			
Yang et al. (47)	China	DTC	66(28/38)	>40	Postsurigical	DNA sequencing	√				√	√				√	√							
Jin et al. (48)	China	PTC	653(150/503)	46.5 ± 12.4	Postsurigical	DNA sequencing	√				√	√				√	√	√	√				√	√
Lee et al. (49)	Korea	PTC	242(56/187)	48.4 ± 13.9	FNAB	DNA sequencing	√				√	√				√	√	√	√		√			√
Song et al. (50)	Korea	PTC, DTC	551(79/472)	>40	Postsurigical	PCR	√	√			√	√	√			√	√	√	√	√	√	√		
Sun et al. (51)	China	PTC	455(124/331)	41.1 ± 11.80	Postsurigical	DNA sequencing	√				√	√				√	√	√						√
Gandolfi (52)	Italy	PTC	121(38/83)	16-90	Postsurigical	DNA sequencing	√				√	√				√	√	√		√			√	
Liu et al. (53)	China	PTC, FTC	408(80/328)	54.89 ± 16.17, 43.73 ± 13.23	Postsurigical	DNA sequencing	√				√	√				√	√	√	√					
Xing et al. (54)	USA	PTC	507(142/365)	45.9 ± 14.0	Postsurigical	PCR and DNA sequencing	√				√	√				√	√	√	√	√	√		√	√
Liu et al. (55)	China	PTC, FTC	367(80/287)	44.78 ± 13.65	Postsurigical	PCR	√				√	√				√	√	√	√					
Henderson et al. (56)	USA	PTC	54(19/35)	–	Postsurigical	PCR and DNA sequencing			√		√			√		√	√	√		√				

PTC, papillary thyroid cancer; FNMTC, familial nonmedullary thyroid carcinoma; FTC, follicular thyroid cancer; DTC, differentiated thyroid cancer.

**Figure 2:**
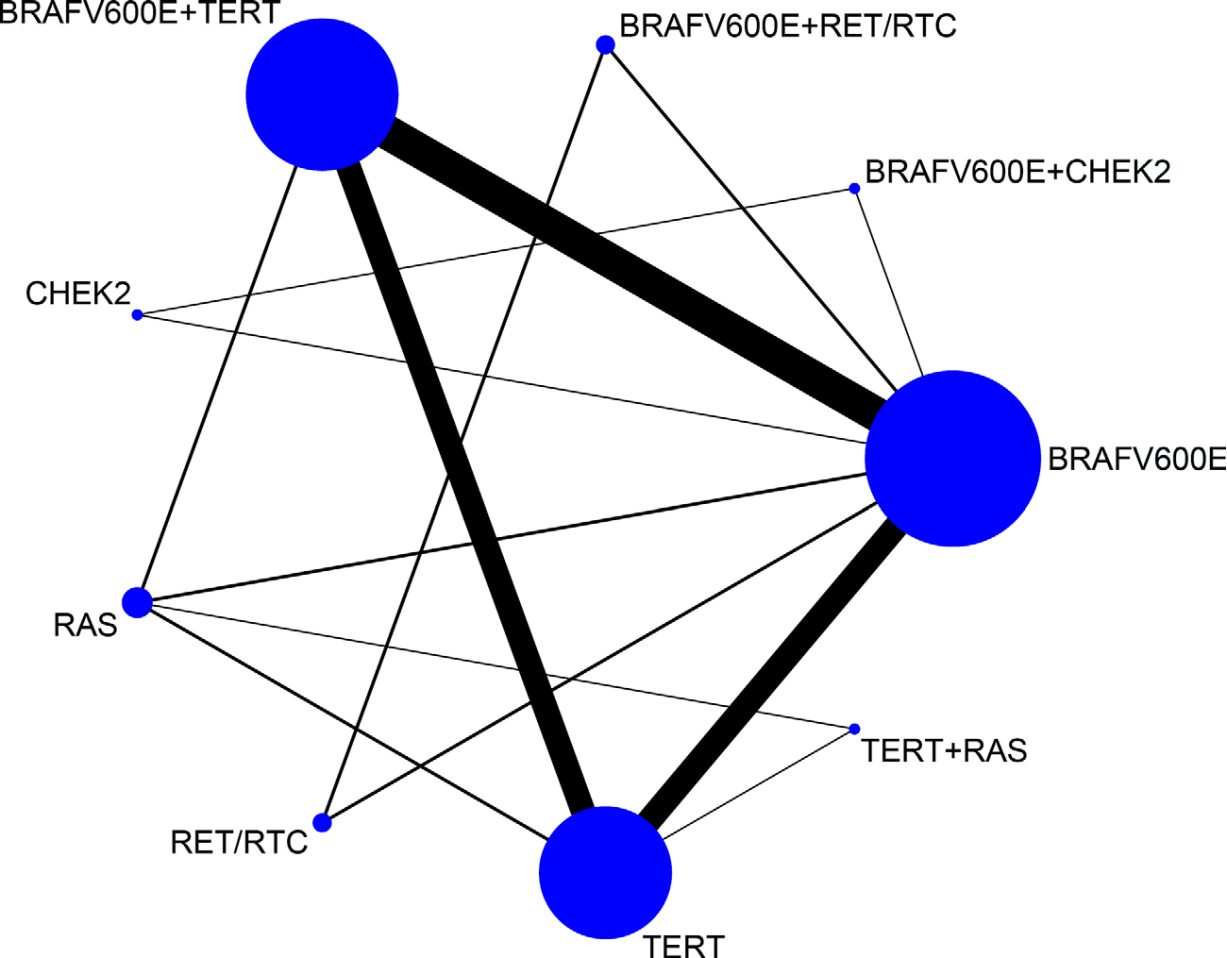
Network meta-analysis plots relating to the eligible comparisons of lymph node metastasis outcomes. Each circular node represents a coexistent or single genetic mutation. The circle size is proportional to the total number of patients with thyroid carcinoma, while the line width is proportional to the number of studies used in the head-to-head comparisons.

We next evaluated four pairs of coexistent genetic mutations: *BRAF^V600E^* + *TERT*, *BRAF^V600E^* + *CHEK2*, *TERT* + *RAS*, and *BRAF^V600E^* + *RET/PTC*, in addition to four isolated genetic mutations involving the same signaling proteins: *BRAF^V600E^*, *TERT*, *RAS*, *CHEK2*, and *RET/PTC*. **Figure 2** shows the network of eligible comparisons for lymph node metastasis. According to the meta-analysis plots, circles represent a coexistent or single genetic mutation. Circle size is proportional to the total number of patients with thyroid carcinoma, while the line width is proportional to the number of studies used in the head-to-head comparisons. The most common coexisting and single genetic mutation comparisons, which made a large contribution to each network estimations, were high frequency *BRAF* ^V600E^ + *TERT* versus *BRAF* ^V600E^; and *BRAF* ^V600E^ + *TERT* versus *TERT*. CASP scales indicated that the 26 selected studies were of adequate quality (**Supplementary Table 2**).

### TC-Based Network Meta-Analysis: Primary Outcomes

**Table 2** show the network meta-analysis results for the primary outcomes, including disease stage, lymph node metastasis, extrathyroidal extension, and distant metastasis. In the evaluation of disease stage (32, 33, 36–39, 44, 45, 47–56), incorporated nine active mutant arms from 18 of the selected studies. Compared with wild-type, *BRAF^V6000E^* + *TERT* mutations ranked highest with significant differences (OR = 5.74, 95% CrI: 3.09–10.66), followed by *BRAF^V6000E^* + *CHEK2* (10.66, 2.10–54.11), *TERT* + *RAS*, *BRAF^V6000E^* + *RET/PTC*, *TERT*, *RET/PTC*, *BRAF^V6000E^*, *CHEK2*, and *RAS*. For the lymph node metastasis outcome form 23 studies (31–42, 44, 45, 48–56), *BRAF* ^V6000E^ + *TERT* also ranked highest, followed by *RET/PTC*, *BRAF^V6000E^*, *BRAF^V6000E^* + *RET/PTC*, *TERT*, *CHEK2*, *TERT*+*RAS*, *BRAF^V6000E^* + *CHEK2*, and *RAS*. Comparisons between the no molecular markers yielded significant result, although both were accompanied by a very low GRADE score.

**Table 2:** Network meta-analysis results for the outcomes in thyroid carcinoma.

Outcomes		Molecular markers	Mutations type	OR(95% CrI)	SUCRA(%)	Rank
Primary outcomes	Disease stage	Coexistent mutations	*BRAF^V600E^+TERT*	5.74 (3.09,10.66)*	89.4	1
			*TERT+RAS*	20.92 (1.93,227.08)*	74.9	3
			*BRAF^V600E^+RET/PTC*	7.55 (0.64,88.87)	64.3	4
			*BRAF^V600E^+CHEK2*	10.66 (2.10,54.11)*	85.7	2
		Single mutation	*BRAF^V600E^*	1.24 (0.74,2.08)	21.6	7
			*TERT*	2.15 (0.95,4.85)	58.1	5
			*RAS*	0.67 (0.15,2.93)	19.7	9
			*RET/PTC*	3.05 (0.06,163.93)	27.8	6
			*CHEK2*	2.06 (0.20,20.81)	21.0	8
	Lymph node metastasis	Coexistent mutations	*BRAF^V600E^+TERT*	1.62 (0.97,2.70)	89.5	1
			*TERT+RAS*	1.38 (0.14,13.61)	35.6	7
			*BRAF^V600E^+RET/PTC*	3.91 (0.37,41.10)	58.6	4
			*BRAF^V600E^+CHEK2*	1.08 (0.18,6.33)	24.3	8
		Single mutation	*BRAF^V600E^*	1.24 (0.80,1.93)	65.5	3
			*TERT*	0.88 (0.46,1.68)	51.1	5
			*RAS*	0.37 (0.08,1.79)	10.5	9
			*RET/PTC*	18.21 (0.44,748.49)	70.6	2
			*CHEK2*	1.77 (0.27,11.50)	43.6	6
	Extrathyroidal extension	Coexistent mutations	*BRAF^V600E^+TERT*	5.74 (4.06,8.10)*	85.2	1
			*TERT+RAS*	2.58 (0.47,14.16)	50.6	4
			*BRAF^V600E^+CHEK2*	1.48 (0.63,3.47)	40.5	5
		Single mutation	*BRAF^V600E^*	1.80 (1.41,2.30)*	78.5	2
			*TERT*	1.72 (1.10,2.68)*	75.9	3
			*RAS*	0.88 (0.40,1.94)	36.3	6
			*CHEK2*	0.60 (0.13,2.67)	30.1	7
	Distant metastasis	Coexistent mutations	*BRAF^V600E^+TERT*	7.86 (3.46,17.84)*	85.0	2
			*TERT+RAS*	39.84 (5.23,303.73)*	93.4	1
			*BRAF^V600E^+RET/PTC*	54.02 (1.37,2124.33)*	55.0	4
			*BRAF^V600E^+CHEK2*	86.43 (0.09,78676.88)	51.6	5
		Single mutation	*BRAF^V600E^*	0.67 (0.29,1.58)	22.9	9
			*TERT*	6.56 (2.24,19.23)*	63.0	3
			*RAS*	3.54 (0.60,21.00)	27.7	8
			*RET/PTC*	36.16 (0.25,5177.90)	47.6	6
			*CHEK2*	6.34 (0.06,680.43)	31.5	7
Secondary outcomes	Tumor recurrence	Coexistent mutations	*BRAF^V600E^+TERT*	7.21 (3.59,14.47)*	91.1	1
			*TERT+RAS*	92.47 (0.08,106876.03)	41.2	4
			*BRAF^V600E^+CHEK2*	48.02 (0.03,67332.82)	34.7	5
		Single mutation	*BRAF^V600E^*	1.58 (0.91,2.77)	61.7	3
			*TERT*	2.67 (1.00,7.15)*	75.4	2
			*RAS*	43.64 (0.04,47930.52)	33.3	7
			*CHEK2*	44.51 (0.03,70519.05)	34.5	6
	Mortality	Coexistent mutations	*BRAF^V600E^+TERT*	9.00 (3.03,26.74)*	82.3	2
			*TERT+RAS*	29.85 (2.36,378.42)*	86.5	1
			*BRAF^V600E^+CHEK2*	95.18 (0.04,225987.43)	57.6	3
		Single mutation	*BRAF^V600E^*	0.85 (0.29,2.46)	24.5	7
			*TERT*	3.54 (0.87,14.36)	27.6	6
			*RAS*	3.69 (0.02,610.95)	38.6	5
			*CHEK2*	88.08 (0.04,209091.33)	55.6	4
	Invasion of the thyroid capsule	Coexistent mutations	*BRAF^V600E^+TERT*	3.11 (1.95,4.95)*	78.2	1
			*BRAF^V600E^+CHEK2*	1.13 (0.24,5.36)	26.8	6
		Single mutation	*BRAF^V600E^*	1.38 (0.99,1.92)	57.1	3
			*TERT*	1.20 (0.00,757.26)	53.3	5
			*RAS*	1.30 (0.17,10.14)	55.1	4
			*CHEK2*	2.55 (1.49,4.37)*	62.8	2
	Multiplicity	Coexistent mutations	*BRAF^V600E^+TERT*	1.28 (0.93,1.76)	57.0	2
			*BRAF^V600E^+CHEK2*	0.97 (0.44,2.15)	41.1	5
		Single mutation	*BRAF^V600E^*	1.01 (0.84,1.22)	55.4	4
			*TERT*	0.81 (0.50,1.29)	35.9	6
			*RAS*	1.37 (0.20,9.48)	79.6	1
			*CHEK2*	1.30 (0.44,3.80)	56.6	3

*significant difference.

For the evaluation of extrathyroidal extension, 16 studies (7 active arms; **Table 2**; (31–34, 36–38, 40, 44, 45, 48–50, 53–55) were included. Of all molecular markers compared with wild-type, *BRAF^V6000E^* + *TERT* ranked highest (5.74, 4.06-8.10), followed by *BRAF^V6000E^* (1.80, 1.41-2.30), *TERT* (1.72, 1.10–2.68), *TERT* + *RAS*, *BRAF^V6000E^* + CHEK2, *RAS*, and CHEK2. In the analysis of distant metastasis (only eight studies, nine active arms; (32, 36, 44, 45, 50, 52, 54, 56), the following mutations were observed in ascending order: *TERT* + *RAS* ranked highest, followed by *BRAF^V6000E^* + *TERT*, *BRAF^V6000E^*, *TERT*, and *RAS*. These above results imply that the coexistence of *BRAF^V6000E^* + *TERT* mutations predicted a worse prognosis for disease stage, extrathyroidal extension in TC patients with significant differences.

### TC-Based Network Meta-Analysis: Secondary Outcomes

We next evaluated the secondary outcomes: tumor recurrence, mortality, invasion of the thyroid capsule and multiplicity (**Table 2**). With regards to tumor recurrence, the coexistence of *BRAF^V6000E^* + *TERT* mutations still ranked the highest in tumor recurrence (eight studies, seven active arms; (32, 36, 44–46, 49, 50, 54), with significant results (7.21, 3.59–14.47) followed by *TERT* (2.67, 1.00–7.15), *BRAF^V6000E^*, *TERT* + *RAS*, *BRAF^V6000E^* + *CHEK2*, and *CHEK2*. With respect to mortality rate [five studies, seven active arms; (36, 43–45, 50)], TERT + *RAS* ranked the highest (29.85, 2.36–378.42), followed by *BRAF^V6000E^* + *TERT* (9.00, 3.03–26.74), *BRAF^V6000E^* + *CHEK2*, *CHEK2*, *RAS*, *TERT*, and *BRAF^V6000E^*. For invasion of the thyroid capsule, nine studies covering six active arms were analyzed [**Table 2**; (32–34, 38, 41, 44, 48, 52, 54)]. The coexisting *BRAF^V6000E^* + *TERT* mutations ranked highest (3.11, 1.95–4.95), followed by *CHEK2* (2.55, 1.49–4.37), *BRAF^V6000E^*, *RAS*, *TERT*, and *BRAF^V6000E^* + *CHEK2.* In terms of multiplicity, the *RAS* mutations ranked highest (13 studies, 6 active arms; (31–33, 35, 36, 40, 41, 44, 45, 48, 49, 51, 54), followed by *BRAF^V6000E^* + *TERT*, *CHEK2*, *BRAF^V6000E^*, *BRAF^V6000E^* + *CHEK2*, and *TERT*. With the two above indicators receiving low GRADE scores.

In summary, the combined mutation of *BRAF^V6000E^* and *TERT* ranked highest in disease stage, lymph node metastasis, extrathyroidal extension, tumor recurrence and invasion of the thyroid capsule; while ranked secondly in distant metastasis, mortality and multiplicity followed by *TERT* + *RAS* or *RAS* alone. This inconsistency may be due to that the research object is all types of TC. We limited the research object to PTC and observed the efficacy of co-mutation of genes in prognosis.

### PTC-Based Network Meta-Analysis: Primary Outcomes

We found that the *BRAF^V600E^* + *TERT* coexistent mutations ranked highest among the majority of studied outcomes. In order to assess study accuracy, we subsequently performed a network meta-analysis of PTC published research (n = 21), by evaluating the role of *BRAF^V600E^* + *TERT* co-mutations in tumor invasion and recurrence.

**Figure 3:**
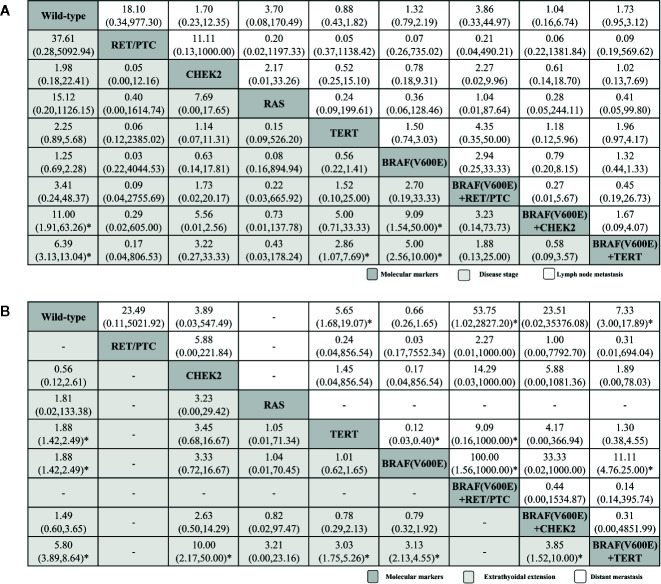
Histopathological feature profiles for the disease stage and lymph node metastasis **(A)**, extrathyroidal extension, and distant metastasis **(B)** based PTC outcomes. From left to right, molecular markers for disease stage and lymph node metastasis **(A)**, extrathyroidal extension and distant metastasis **(B)** are ranked by mean rank and SUCRA score. Information relating to the ORs and 95% CrI is listed in the column, with the rows displaying molecular marker identity. OR values higher than 1 favor the column-defining treatment (i.e., the left-most in order), indicating histopathological features associated with a worse prognosis. To obtain OR values for comparisons in the opposite direction, reciprocals should be taken. *Statistical significance.

**Figure 3** summarizes four typical outcomes with respect to PTC metastasis, invasion and recurrence, including disease stage and lymph node metastasis (**A**), extrathyroidal extension, and distant metastasis (**B**). Data relating to PTC disease stage were available from 14 of the selected publications (eight active arms; (32, 33, 36–39, 45, 48–52, 54, 56). For all genetic mutant arms, the *BRAF^V600E^* + TERT coexistent mutation ranked highest with significant different compared with wild-type (6.39, 3.13–13.04), followed by *BRAF^V6000E^* + *CHEK2* (11.00, 1.91–63.26), *BRAF^V6000E^* + *RET/PTC*, *BRAF^V6000E^*, *TERT*, *RAS*, *CHEK2*, and *RET/PTC*. Comparisons between the following molecular markers yielded significant results: *BRAF^V6000E^*+ *TERT* versus *TERT*, *BRAF^V6000E^* + *TERT* and *BRAF^V6000E^*, as well as *BRAF^V6000E^* + *CHEK2* versus *BRAF^V6000E^*. Data observing to PTC lymph node metastasis were available from 20 articles (eight active arms; (31–42, 45, 48–52, 54, 56). The *BRAF^V600E^* + *TERT* coexistent mutation ranked highest, again, with no significant different, followed by *BRAF^V600E^* + *RET/PTC*, *BRAF^V6000E^* + *CHEK2*, *BRAF^V600E^*, *RET/PTC*, *RAS*, *CHEK2*, and *TERT*, with no significant results among all comparisons (**Figure 3A**), and had a very low GRADE score from such above outcomes.

Extrathyroidal extension outcome analysis included data form 13 of the research papers (six active arms; **Figure 3B**; (31–34, 36–38, 40, 45, 48–50, 54). The *BRAF^V600E^* + *TERT* coexistent mutation once again ranked highest (5.80, 3.89–8.64), while followed by *BRAF^V6000E^* (1.88, 1.42–2.49), TERT (1.88, 1.42–2.49), *RAS*, *BRAF^V6000E^* + *CHEK2*, and *CHEK2.* Comparisons between the following molecular markers yielded significant differences: *BRAF^V600E^* + *TERT* versus *BRAF^V600E^*, *BRAF^V600E^* + *TERT* versus *TERT*, *BRAF^V600E^* + *TERT* versus *BRAF^V6000E^* +*CHEK2* and *BRAF^V600E^* + *TERT* versus *CHEK2.* For distant metastasis for seven original researches (seven active arms; **Figure 3B**; (32, 36, 45, 50, 52, 54, 56). *BRAF^V600E^* + *TERT* coexistent mutation ranked highest again (7.33, 3.00–17.89), which is inconsistent with the overall TC result, followed by *BRAF^V600E^* + *RET/PTC* (53.75, 1.02–2927.20), *TERT* (5.65, 1.68–19.07), *BRAF^V6000E^* + *CHEK2*, *RET/PTC*, *CHEK2*, and *BRAF^V600E^*, significant results could be found in *BRAF^V600E^* + *TERT* versus *BRAF^V600E^*. Generally, *BRAF^V600E^* + *TERT* always ranked first in our primary outcomes.

### PTC-Based Network Meta-Analysis: Secondary Outcomes

**Figure 4:**
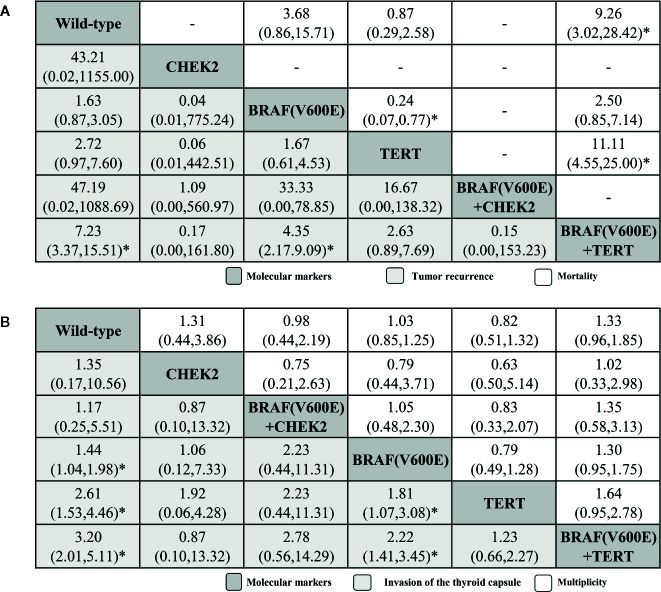
Histopathological feature profiles for the tumor recurrence and mortality **(A)**, invasion of the thyroid capsule and multiplicity **(B)** based PTC outcomes. From left to right, molecular markers for tumor recurrence and mortality **(A)**, invasion of the thyroid capsule and multiplicity **(B)** are ranked by mean rank and SUCRA score. Information relating to the ORs and 95% CrI is listed in the column, with the rows displaying molecular marker identity. OR values higher than 1 favor the column-defining treatment (i.e., the left-most in order), indicating histopathological features associated with a worse prognosis. To obtain ORs for comparisons in the opposite direction, reciprocals should be taken. *Significant results.

The tumor recurrence results were obtained from six of the selected publications (five arms; **Figure 4A**; (32, 36, 45, 49, 50, 54) and showed that the *BRAF^V600E^* + TERT coexistent mutation also ranked highest (7.23, 3.37–15.51), followed by *BRAF^V600E^* (4.35, 2.17–9.09), *TERT*, *CHEK2*, and *BRAF^V6000E^* + *CHEK2.* For mortality outcome from four studies (three arms; **Figure 4A**; (36, 43, 45, 50), *BRAF^V600E^* + *TERT* ranked first (9.26, 3.02–28.42), followed by *BRAF^V6000E^* and *TERT*. Significance tested in *BRAF^V600E^* + *TERT* versus *TERT* and *TERT* versus *BRAF^V600E^* groups.

The invasion of the thyroid capsule results were from only eight research papers (five active arms; (32–34, 38, 41, 48, 52, 54). The *BRAF^V600E^* + *TERT* coexistent mutation again ranked highest (3.20, 2.01–5.11), followed by *TERT* (2.61, 1.53–4.46), *BRAF^V600E^* (1.44, 1.04–1.98), *BRAF^V6000E^* + *CHEK2*, and *CHEK2.* Comparisons between the following molecular markers yielded significant results: *BRAF^V6000E^* + *TERT* versus *BRAF^V6000E^* and *TERT* versus *BRAF^V6000E^*. For multiplicity outcome (five active arms; (31–36, 40, 41, 45, 48, 49, 51, 54), ranking order were *BRAF^V6000E^* + *TERT*, *BRAF^V6000E^*, *CHEK2*, *BRAF^V6000E^* + *CHEK2*, and *TERT*.

In summary, the co-mutation of *BRAF^V600E^* + *TERT* was more significant in PTC, which always ranking first. And the significant results were found in the outcomes disease stage, extrathyroid extension, distant metastasis, tumor recurrence, mortality, and invasion of thyroid capsule. Which means the *BRAF^V600E^* + *TERT* co-mutation plays an important role in the invasion and recurrence of TC, and above all, PTC.

## Discussion

Our systematic review and network meta-analysis, evaluating the coexistence of genetic mutations as a valuable means of predicting the histopathological features associated with TC prognosis, included 8388 patients from 26 quality original research articles. Firstly, among the primary outcomes, coexistence of the *BRAF^V600E^* + *TERT* mutations ranked: i) highest in the disease stage and extrathyroidal extension outcomes; ii) second in distant metastasis and mortality outcome. Furthermore, the *BRAF^V600E^* + *TERT* co-mutation ranked highest in all of the following secondary outcomes: tumor recurrence, mortality, invasion of thyroid capsule; and ranked second in multiplicity outcome. Moreover, on performing another network meta-analysis of the outcomes related to patients with PTC, we noticed that the coexistent *BRAF^V600E^* + *TERT* mutation also ranked highest in all of the outcomes. Our research complies with the PRISMA guidelines and was registered with the PROSPERO cooperative, in order to assure that the study is both systematic and gradual in nature.

Of the eight outcomes (primary outcomes: lymph node metastasis, disease stage, distant metastatis, and extrathyroidal extension; and secondary outcomes: tumor recurrence, mortality, invasion of the thyroid capsule, and multiplicity) analyzed, the coexistent *BRAF^V600E^* + *TERT* mutation ranked highest five times (**Table 2**), demonstrating that it has a profound impact on the histopathological features associated with a worse prognosis. Giorgenon et al. documented a significant association between the dual TERT*p/BRAF^V600E^* mutation and advanced stage, compared with the control group that was negative for two mutations (33), consistent with our results. Kim and colleagues concluded that, compared with the presence of a single mutation, concomitant *TERT* and *BRAF* mutations worsened the survival rate of papillary cancer patients (57). Similarly, in our network meta-analysis of PTC patients, coexistent mutations always ranked highest as molecular markers of invasion, progression and recurrence (**Figures 3** and **4**). Our findings are in keeping with work by Jin et al., who demonstrated a significant role of *BRAF^V600E^* and *TERT* promoter mutations in PTC, which is particularly aggressive in cases when the two mutations coexist (48).

We also found an indication of the *TERT* promoter mutation, either alone or in combination with the *BRAF^V600E^* mutation (which ranked second followed by *TERT* promoter single mutation), having a certain effect on the invasion of thyroid capsule outcome (**Figure 4**). This observation confirms that the *TERT* promoter mutation with or without *BRAF^V600E^* mutation represents an independent prognostic factor for poor prognosis. Similar results were found in the study by Kim et al., in which they concluded that concomitant *TERT* and *BRAF* mutations worsened the survival rate of patients with papillary cancer (57). Moreover, a study by Melo and colleagues, reported that distant metastases were enriched for *TERTp* mutations but depleted in *BRAF* mutations. *TERTp* mutations may play a role in distant metastases, which is consistent with our results (58).

Our research proves that when the research type is TC, two outcome indicators (distant metastasis and mortality) showed that *TERT*+*RAS* ranked first from network meta-analysis, and another outcome indicator (multiplicity) showed that *RAS* ranked first. The above results suggest that *RAS* mutations may also be one of the main reasons affecting long-term prognosis. These analyses were performed for TC in general, and similar study by Bellevicine C’s research found that *RAS* was strictly related to the risk of malignancy of TC (59), and previous studies have demonstrated that the presence of *RAS* mutations in a thyroid nodule provides evidence for neoplasia (60). Thus, we made a conclusion that *BRAF^V600E^*, *TERT*, and *RAS* triple mutations may herald a worse prognosis. For research type, which is limited to PTC, we confirm that coexistent *BRAF^V600E^* + *TERT* genetic mutations are the best predictors of poor TC prognosis and have the highest impact on the tumor progression, invasion, and recurrence in patients with PTC.

Despite the systematic nature of our work, there are several limitations to this study. Firstly, we only performed network meta-analysis. Although the results included head-to-head results, there was no direct comparison between all combination of mutations (e.g., *BRAF^V600E^* + *TERT* versus *BRAF^V600E^* + *RET/PTC*), which could have only been obtained through inaccurate indirect comparisons. In addition, our result GRADE scores ranged from low and very low, due to the exclusion of randomized control trials, and the inclusion of indirect comparisons. Moreover, the ‘no mutation’ controls were different for each group. For instance, a given study may have only comprised data relating to the *BRAF^V600E^* + *TERT* genetic co-mutations and *BRAF^V600E^* and *TERT* single mutations while not considering *RAS* mutation, *CHEK2* mutation, or *RET/PTC* rearrangements, which may have an impact on the overall outcomes, which is also the reason for our limitations. In such cases, it is possible that the no mutation group actually included other kinds of mutations, which may have a certain impact on the overall results. What’s more, AJCC staging system 8^th^ edition begins to be used internationally from Jan 2019, and only two studies using the new edition AJCC staging system (32, 33), which maybe also a limitation of our research. Last but not least, we were only able to select 21 studies relating to PTC, and were therefore limited by patient numbers. We decided not to include FTC studies within the PTC analysis group for the sake of increasing our samples size, as this would limit the accuracy of results. Notwithstanding these limitations, our network meta-analysis is the first comprehensive study to document the effect of genetic co-mutations on the prognosis of TC patients.

The synergistic impact of the *BRAF^V600E^* + *TERTp* co-mutations on the invasiveness and progression of PTC may be explained in part by increased *TERT* expression, which may result from the *BRAF*-induced up-regulation of several E26 transcription factors (36). Coincidentally, another study has claimed that the *BRAF^V600E^*-activated MAPK pathway may selectively up-regulate mutant *TERT* proteins, thus promoting cooperative oncogenesis (61). The *BRAF* gene belongs to the *RAF* gene family. It is a downstream signaling molecule of *RET* and *RAS*. It can encode a silk/threonine-specific kinase and is the most effective activator in the MAPK/extracellular regulated protein kinases (ERK) pathway. *RAF* is also an activator with the strongest kinase activity in the family, continuous activation of the MAPK signaling pathway, and the slenderness leads to abnormal cell proliferation, differentiation, uncontrolled cell cycle, and in circulation, thereby forming tumors. So from the perspective of mechanism, the results we have obtained are valid.

In this systematic review and network meta-analysis, we have identified clinically-important differences between the histopathological prognostic features associated with coexistent versus single mutations in TC. We found that the *BRAF^V600E^* + *TERT* co-mutations predicted poor histopathological prognosis, including progression, invasion, and metastasis, especially in PTC. Also, further research should related to potentially important features such as molecular profile and clinical outcome. For the overall TC, the *BRAF^V600E^* + *TERT* + *RAS* triple mutations may have a greater impact on the prognosis.

## Data Availability Statement

All datasets analyzed for this study are included in the article/**Supplementary Material**.

## Author Contributions

Conceptualization: ZL. Methodology (data collection): YZ and LW. Statistical analysis: YZ, LW, LZ, XJ, XH, PP, SZ, YW, and JW. Writing (original draft preparation): YZ and LW. Review and editing: ZL. All authors contributed to the article and approved the submitted version.

## Conflict of Interest

The authors declare that the research was conducted in the absence of any commercial or financial relationships that could be construed as a potential conflict of interest.

## Supplementary Material

Supplementary Figure 1 Inconsistency plot for the lymph node metastasis outcome in TC.

Supplementary Figure 2 Funnel plot for the lymph node metastasis outcome in TC.

Supplementary Figure 3 Inconsistency plot for the extrathyroidal extension outcome in TC.

Supplementary Figure 4 Inconsistency plot for the lymph node metastasis outcome in PTC.

Supplementary Table 1 Search strategies.

Supplementary Table 2 Quality assessment of included of studies by Critical Appraisal Skills Programme (CASP) scales.

Supplementary Table 3 Assessment of the quality of evidence using the Grading of Recommendations Assessment.
