# Supplementary figures and images for: The Coexistence of Genetic Mutations in Thyroid Carcinoma Predicts Histopathological Factors Associated With a Poor Prognosis: A Systematic Review and Network Meta-Analysis
Source: Front Oncol. 2020 Nov 3;10:540238. doi: 10.3389/fonc.2020.540238 (PMC7682272; doi:10.3389/fonc.2020.540238)

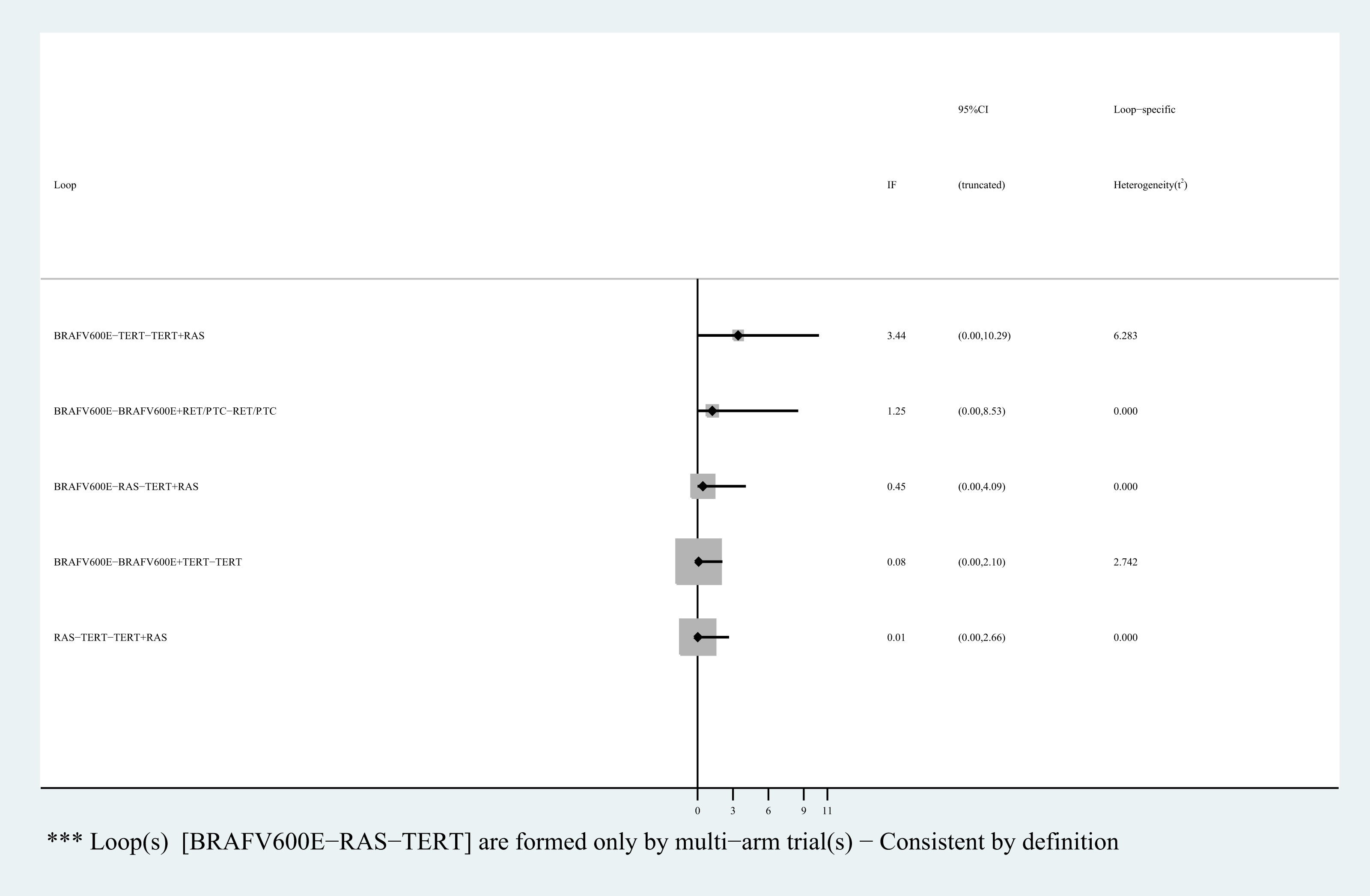

Supplement: Supplementary Figure 1 — Inconsistency plot for the lymph node metastasis outcome in TC. [file Image_1.tif]

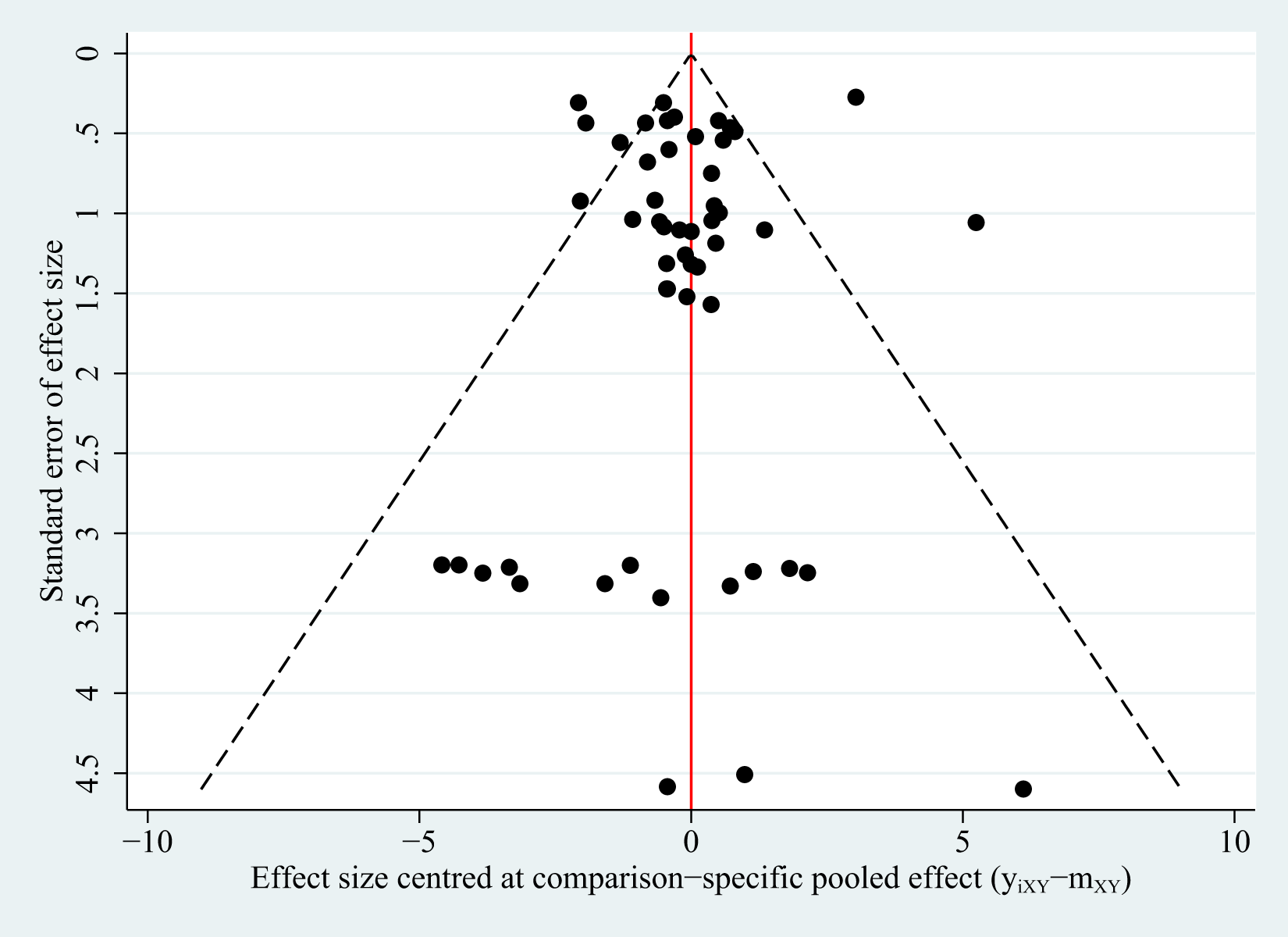

Supplement: Supplementary Figure 2 — Funnel plot for the lymph node metastasis outcome in TC. [file Image_2.tif]

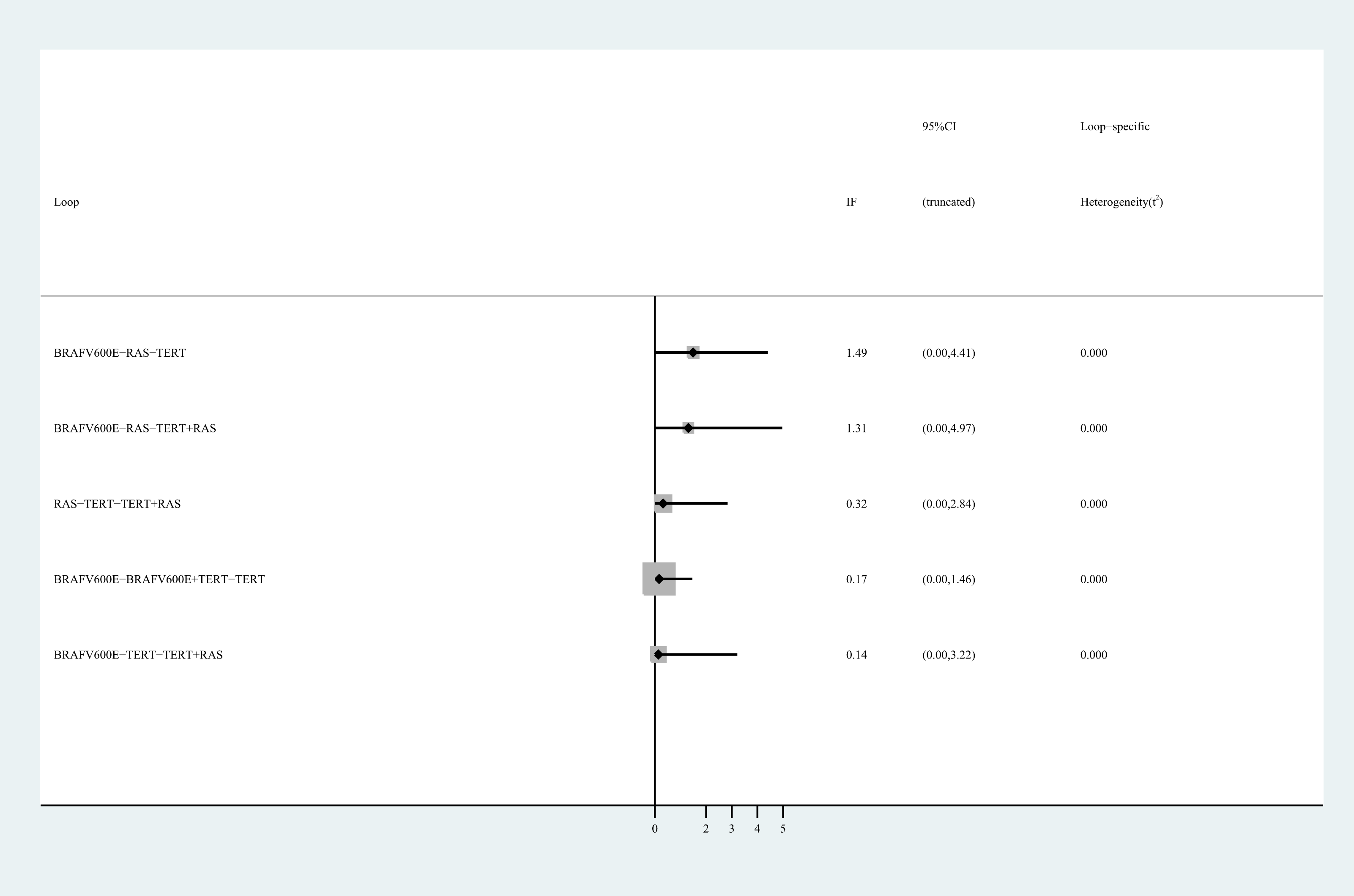

Supplement: Supplementary Figure 3 — Inconsistency plot for the extrathyroidal extension outcome in TC. [file Image_3.tif]

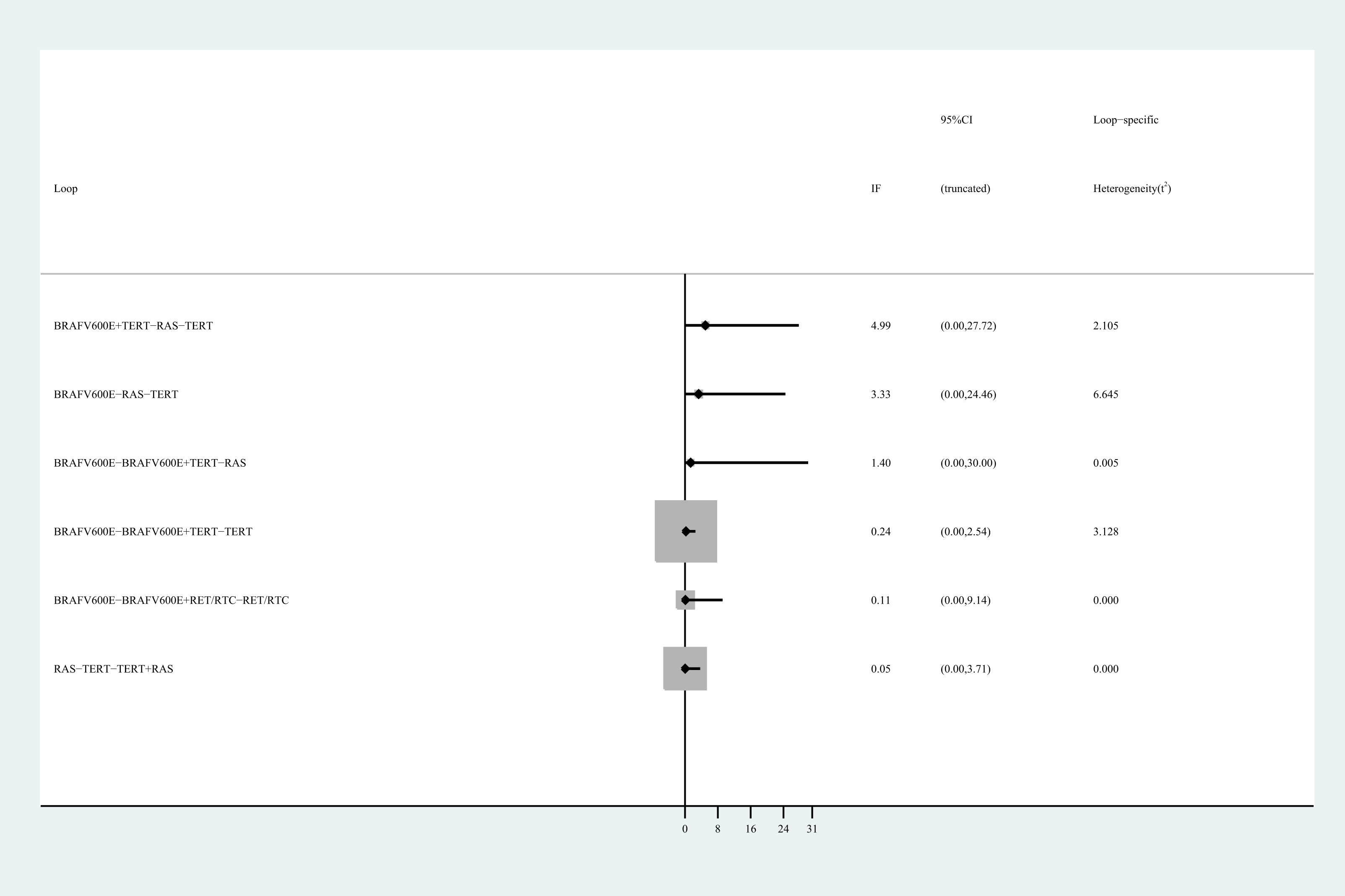

Supplement: Supplementary Figure 4 — Inconsistency plot for the lymph node metastasis outcome in PTC. [file Image_4.tif]
