# Supplementary material for: The Coexistence of Genetic Mutations in Thyroid Carcinoma Predicts Histopathological Factors Associated With a Poor Prognosis: A Systematic Review and Network Meta-Analysis
Source: Front Oncol. 2020 Nov 3;10:540238. doi: 10.3389/fonc.2020.540238 (PMC7682272; doi:10.3389/fonc.2020.540238)
Supplement: Supplementary Table 1 — Search strategies. [file Table_1.doc]

**Supplementary Table 1 Search strategies**

**Search strategies for PubMed**

#1. ("mutation"[MeSH Terms] OR "mutation"[All Fields] OR ("genetic"[All Fields] AND "mutations"[All Fields]) OR "genetic mutations"[All Fields]) AND ("thyroid neoplasms"[MeSH Terms] OR ("thyroid"[All Fields] AND "neoplasms"[All Fields]) OR "thyroid neoplasms"[All Fields] OR ("thyroid"[All Fields] AND "carcinoma"[All Fields]) OR "thyroid carcinoma"[All Fields])

#2. ("mutation"[MeSH Terms] OR "mutation"[All Fields] OR ("genetic"[All Fields] AND "mutations"[All Fields]) OR "genetic mutations"[All Fields]) AND ("thyroid neoplasms"[MeSH Terms] OR ("thyroid"[All Fields] AND "neoplasms"[All Fields]) OR "thyroid neoplasms"[All Fields] OR ("thyroid"[All Fields] AND "cancer"[All Fields]) OR "thyroid cancer"[All Fields])

#3. #1 OR #2

#4. BRAFV600E[All Fields] AND TERT[All Fields] AND ("thyroid neoplasms"[MeSH Terms] OR ("thyroid"[All Fields] AND "neoplasms"[All Fields]) OR "thyroid neoplasms"[All Fields] OR ("thyroid"[All Fields] AND "carcinoma"[All Fields]) OR "thyroid carcinoma"[All Fields])

#5. BRAFV600E[All Fields] AND TERT[All Fields] AND ("thyroid neoplasms"[MeSH Terms] OR ("thyroid"[All Fields] AND "neoplasms"[All Fields]) OR "thyroid neoplasms"[All Fields] OR ("thyroid"[All Fields] AND "cancer"[All Fields]) OR "thyroid cancer"[All Fields])

#6. ("proto-oncogene proteins b-raf"[MeSH Terms] OR ("proto-oncogene"[All Fields] AND "proteins"[All Fields] AND "b-raf"[All Fields]) OR "proto-oncogene proteins b-raf"[All Fields] OR "b raf"[All Fields]) AND ("proto-oncogenes"[MeSH Terms] OR "proto-oncogenes"[All Fields] OR ("proto"[All Fields] AND "oncogene"[All Fields]) OR "proto oncogene"[All Fields]) AND ("telomerase"[MeSH Terms] OR "telomerase"[All Fields] OR ("telomerase"[All Fields] AND "reverse"[All Fields] AND "transcriptase"[All Fields]) OR "telomerase reverse transcriptase"[All Fields]) AND ("thyroid neoplasms"[MeSH Terms] OR ("thyroid"[All Fields] AND "neoplasms"[All Fields]) OR "thyroid neoplasms"[All Fields] OR ("thyroid"[All Fields] AND "carcinoma"[All Fields]) OR "thyroid carcinoma"[All Fields])

#7. ("proto-oncogene proteins b-raf"[MeSH Terms] OR ("proto-oncogene"[All Fields] AND "proteins"[All Fields] AND "b-raf"[All Fields]) OR "proto-oncogene proteins b-raf"[All Fields] OR "b raf"[All Fields]) AND ("proto-oncogenes"[MeSH Terms] OR "proto-oncogenes"[All Fields] OR ("proto"[All Fields] AND "oncogene"[All Fields]) OR "proto oncogene"[All Fields]) AND ("telomerase"[MeSH Terms] OR "telomerase"[All Fields] OR ("telomerase"[All Fields] AND "reverse"[All Fields] AND "transcriptase"[All Fields]) OR "telomerase reverse transcriptase"[All Fields]) AND ("thyroid neoplasms"[MeSH Terms] OR ("thyroid"[All Fields] AND "neoplasms"[All Fields]) OR "thyroid neoplasms"[All Fields] OR ("thyroid"[All Fields] AND "cancer"[All Fields]) OR "thyroid cancer"[All Fields])

#8. #4 OR #5 OR #6 OR #7

#9.TERT[All Fields] AND RAS[All Fields] AND ("thyroid neoplasms"[MeSH Terms] OR ("thyroid"[All Fields] AND "neoplasms"[All Fields]) OR "thyroid neoplasms"[All Fields] OR ("thyroid"[All Fields] AND "carcinoma"[All Fields]) OR "thyroid carcinoma"[All Fields])

#10.TERT[All Fields] AND RAS[All Fields] AND ("thyroid neoplasms"[MeSH Terms] OR ("thyroid"[All Fields] AND "neoplasms"[All Fields]) OR "thyroid neoplasms"[All Fields] OR ("thyroid"[All Fields] AND "cancer"[All Fields]) OR "thyroid cancer"[All Fields])

#11.("telomerase"[MeSH Terms] OR "telomerase"[All Fields] OR ("telomerase"[All Fields] AND "reverse"[All Fields] AND "transcriptase"[All Fields]) OR "telomerase reverse transcriptase"[All Fields]) AND RAS[All Fields] AND ("thyroid neoplasms"[MeSH Terms] OR ("thyroid"[All Fields] AND "neoplasms"[All Fields]) OR "thyroid neoplasms"[All Fields] OR ("thyroid"[All Fields] AND "cancer"[All Fields]) OR "thyroid cancer"[All Fields])

#12.("telomerase"[MeSH Terms] OR "telomerase"[All Fields] OR ("telomerase"[All Fields] AND "reverse"[All Fields] AND "transcriptase"[All Fields]) OR "telomerase reverse transcriptase"[All Fields]) AND RAS[All Fields] AND ("thyroid neoplasms"[MeSH Terms] OR ("thyroid"[All Fields] AND "neoplasms"[All Fields]) OR "thyroid neoplasms"[All Fields] OR ("thyroid"[All Fields] AND "carcinoma"[All Fields]) OR "thyroid carcinoma"[All Fields])

#13. #9 OR #10 OR #11 OR #12

#14. BRAF[All Fields] AND RET/PTC[All Fields] AND ("thyroid neoplasms"[MeSH Terms] OR ("thyroid"[All Fields] AND "neoplasms"[All Fields]) OR "thyroid neoplasms"[All Fields] OR ("thyroid"[All Fields] AND "carcinoma"[All Fields]) OR "thyroid carcinoma"[All Fields])

#15.BRAF[All Fields] AND RET/PTC[All Fields] AND ("thyroid neoplasms"[MeSH Terms] OR ("thyroid"[All Fields] AND "neoplasms"[All Fields]) OR "thyroid neoplasms"[All Fields] OR ("thyroid"[All Fields] AND "cancer"[All Fields]) OR "thyroid cancer"[All Fields])

#16.("proto-oncogene proteins b-raf"[MeSH Terms] OR ("proto-oncogene"[All Fields] AND "proteins"[All Fields] AND "b-raf"[All Fields]) OR "proto-oncogene proteins b-raf"[All Fields] OR "b raf"[All Fields]) AND ("proto-oncogenes"[MeSH Terms] OR "proto-oncogenes"[All Fields] OR ("proto"[All Fields] AND "oncogene"[All Fields]) OR "proto oncogene"[All Fields]) AND ("J Ration Emot Cogn Behav Ther"[Journal] OR "ret"[All Fields]) AND ("thyroid cancer, papillary"[MeSH Terms] OR ("thyroid"[All Fields] AND "cancer"[All Fields] AND "papillary"[All Fields]) OR "papillary thyroid cancer"[All Fields] OR ("papillary"[All Fields] AND "thyroid"[All Fields] AND "cancer"[All Fields]))

#17.#14 OR #15 OR #16

#18. BRAFV600E[All Fields] AND TERT[All Fields] AND RAS[All Fields] AND RET/PTC[All Fields]

#19.("proto-oncogene proteins b-raf"[MeSH Terms] OR ("proto-oncogene"[All Fields] AND "proteins"[All Fields] AND "b-raf"[All Fields]) OR "proto-oncogene proteins b-raf"[All Fields] OR "b raf"[All Fields]) AND proto-oncogeneV600E[All Fields] AND ("telomerase"[MeSH Terms] OR "telomerase"[All Fields] OR ("telomerase"[All Fields] AND "reverse"[All Fields] AND "transcriptase"[All Fields]) OR "telomerase reverse transcriptase"[All Fields]) AND RAS[All Fields] AND RET/Papillary[All Fields] AND ("thyroid neoplasms"[MeSH Terms] OR ("thyroid"[All Fields] AND "neoplasms"[All Fields]) OR "thyroid neoplasms"[All Fields] OR ("thyroid"[All Fields] AND "cancer"[All Fields]) OR "thyroid cancer"[All Fields])

#20. #18 OR#19

#21. #3 and #8 and #13 and #17 and #20

**Search strategies for EMbase**

#1. genetic mutations thyroid carcinoma.mp. [mp=ti, ab, tx, ct, sh, ot, nm, hw, fx, kf, ox, px, rx, an, ui, sy]

#2. genetic mutations thyroid cancer.mp. [mp=ti, ab, tx, ct, sh, ot, nm, hw, fx, kf, ox, px, rx, an, ui, sy]

#3. BRAFV600E TERT thyroid carcinoma.mp. [mp=ti, ab, tx, ct, sh, ot, nm, hw, fx, kf, ox, px, rx, an, ui, sy]

#4. BRAFV600E TERT thyroid cancer.mp. [mp=ti, ab, tx, ct, sh, ot, nm, hw, fx, kf, ox, px, rx, an, ui, sy]

#5. B-Raf proto-oncogene TERT thyroid carcinoma.mp. [mp=ti, ab, tx, ct, sh, ot, nm, hw, fx, kf, ox, px, rx, an, ui, sy]

#6. B-Raf proto-oncogene TERT thyroid cancer.mp. [mp=ti, ab, tx, ct, sh, ot, nm, hw, fx, kf, ox, px, rx, an, ui, sy]

#7. B-Raf proto-oncogene Telomerase Reverse Transcriptase thyroid carcinoma.mp. [mp=ti, ab, tx, ct, sh, ot, nm, hw, fx, kf, ox, px, rx, an, ui, sy]

#8. B-Raf proto-oncogene Telomerase Reverse Transcriptase thyroid cancer.mp. [mp=ti, ab, tx, ct, sh, ot, nm, hw, fx, kf, ox, px, rx, an, ui, sy]

#9.TERT RAS thyroid carcinoma.mp. [mp=ti, ab, tx, ct, sh, ot, nm, hw, fx, kf, ox, px, rx, an, ui, sy]

#10.TERT RAS thyroid cancer.mp. [mp=ti, ab, tx, ct, sh, ot, nm, hw, fx, kf, ox, px, rx, an, ui, sy]

#11. Telomerase Reverse Transcriptase RAS thyroid carcinoma.mp. [mp=ti, ab, tx, ct, sh, ot, nm, hw, fx, kf, ox, px, rx, an, ui, sy]

#12. Telomerase Reverse Transcriptase RAS thyroid cancer.mp. [mp=ti, ab, tx, ct, sh, ot, nm, hw, fx, kf, ox, px, rx, an, ui, sy]

#13. BRAF RET/PTC thyroid carcinoma.mp. [mp=ti, ab, tx, ct, sh, ot, nm, hw, fx, kf, ox, px, rx, an, ui, sy]

#14. BRAF RET/PTC thyroid cancer.mp. [mp=ti, ab, tx, ct, sh, ot, nm, hw, fx, kf, ox, px, rx, an, ui, sy]

#15. B-Raf proto-oncogene RET/PTC thyroid carcinoma.mp. [mp=ti, ab, tx, ct, sh, ot, nm, hw, fx, kf, ox, px, rx, an, ui, sy]

#16. B-Raf proto-oncogene thyroid cancer.mp. [mp=ti, ab, tx, ct, sh, ot, nm, hw, fx, kf, ox, px, rx, an, ui, sy]

#17. #1 and #2 and #3 and #4 and #5 and #6 and #7 and #8 and #9 and #10 and #11 and #12 and #13 and #14 and #15 and #16

**Search strategies for Cochrane library**

#1.BRAFV600E, TERT, RAS, RET/PTC, thyroid carcinoma

#2.BRAFV600E, TERT, RAS, RET/PTC, thyroid cancer

#3.B-Raf proto-oncogeneV600E, Telomerase Reverse Transcriptase, RAS, RET/PTC, thyroid carcinoma

#4.B-Raf proto-oncogeneV600E, Telomerase Reverse Transcriptase, RAS, RET/PTC, thyroid cancer

#5. MeSH descriptor

#6. #1 and #2 and #3 and #4 and #5
