# Supplementary material for: The Coexistence of Genetic Mutations in Thyroid Carcinoma Predicts Histopathological Factors Associated With a Poor Prognosis: A Systematic Review and Network Meta-Analysis
Source: Front Oncol. 2020 Nov 3;10:540238. doi: 10.3389/fonc.2020.540238 (PMC7682272; doi:10.3389/fonc.2020.540238)
Supplement: Supplementary Table 2 — Quality assessment of included of studies by Critical Appraisal Skills Programme (CASP) scales. [file Table_2.doc]

**Table S2 Quality assessment of included of studies by Critical Appraisal Skills Programme (CASP) scales.**

| **Study, Year** | **1.Did the study address a clearly focused issue?** | **2.Was the cohort recruited in an acceptable way?** | **3.Was the exposure accurately measured to minimise bias?** | **4.Was the outcome accurately measured to minimise bias?** | **5. (a) Have the authors identified all important confounding factors?** | **5. (b) Have they taken account of the confounding factors in the design and/or analysis?** | **6. (a) Was the follow up of subjects complete enough?** | **6. (b) Was the follow up of subjects long enough?** | **7. What are the results of this study?** | **8. How precise are the results?** | **9. Do you believe the results?** | **10.Can the results be applied to the local population?** | **11. Do the results of this study fit  with other available  evidence?** | **12. What are the implications of  this study for practice?** |
| --- | --- | --- | --- | --- | --- | --- | --- | --- | --- | --- | --- | --- | --- | --- |
| Colombo C,2019[1] | Yes | Yes | Yes | Yes | Can’t tell | Can’t tell | Can’t tell | Can’t tell | Yes | Can’t tell | Yes | Yes | Yes | Yes |
| Gąsior-Perczak D,2019[2] | Yes | Yes | Yes | Yes | Can’t tell | Can’t tell | Yes | Yes | Yes | Can’t tell | Yes | Yes | Yes | Yes |
| Giorgenon TMV,2019[3] | Yes | Yes | Yes | Yes | Can’t tell | Can’t tell | Yes | Can’t tell | Yes | Can’t tell | Yes | Yes | Yes | Yes |
| Hou X,2019[4] | Yes | Yes | Yes | Yes | Can’t tell | Can’t tell | Can’t tell | Can’t tell | Yes | Can’t tell | Yes | Can’t tell | Yes | Yes |
| Huang M,2019[5] | Yes | Yes | Yes | Yes | Can’t tell | Can’t tell | Can’t tell | Can’t tell | Yes | Can’t tell | Yes | Yes | Yes | Yes |
| Song YS,2019[6] | Yes | Yes | Yes | Yes | Can’t tell | Can’t tell | Can’t tell | Can’t tell | Yes | Can’t tell | Yes | Yes | Yes | Yes |
| Argyropoulou M,2018[7] | Yes | Yes | Yes | Yes | Can’t tell | Can’t tell | Can’t tell | Can’t tell | Yes | Can’t tell | Yes | Can’t tell | Yes | Yes |
| Dai L,2018[8] | Yes | Yes | Yes | Yes | Can’t tell | Can’t tell | Can’t tell | Can’t tell | Yes | Can’t tell | Yes | Yes | Yes | Yes |
| Deng F,2018[9] | Yes | Yes | Yes | Yes | Can’t tell | Can’t tell | Can’t tell | Can’t tell | Yes | Can’t tell | Yes | Yes | Yes | Yes |
| Ren H,2018[10] | Yes | Yes | Yes | Yes | Can’t tell | Can’t tell | No | No | Yes | Can’t tell | Yes | Yes | Yes | Yes |
| Rusinek D,2018[11] | Yes | Yes | Yes | Yes | Can’t tell | Can’t tell | Can’t tell | Can’t tell | Yes | Can’t tell | Yes | Can’t tell | Yes | Yes |
| Zhou D,2018[12] | Yes | Yes | Yes | Yes | Can’t tell | Can’t tell | Yes | Yes | Yes | Can’t tell | Yes | Yes | Yes | Yes |
| Liu R, 2017[13] | Yes | Yes | Yes | Yes | Can’t tell | Can’t tell | Yes | Yes | Yes | Can’t tell | Yes | Can’t tell | Yes | Yes |
| Marques IJ,2017[14] | Yes | Yes | Yes | Yes | Can’t tell | Can’t tell | Can’t tell | Can’t tell | Yes | Can’t tell | Yes | Can’t tell | Yes | Yes |
| Shen X,2017[15] | Yes | Yes | Yes | Yes | Can’t tell | Can’t tell | Yes | Yes | Yes | Can’t tell | Yes | Can’t tell | Yes | Yes |
| Song YS,2017[16] | Yes | Yes | Yes | Yes | Can’t tell | Can’t tell | Yes | Yes | Yes | Can’t tell | Yes | Yes | Yes | Yes |
| Yang X,2017[17] | Yes | Yes | Yes | Yes | Can’t tell | Can’t tell | Yes | Yes | Yes | Can’t tell | Yes | Yes | Yes | Yes |
| Jin L,2016[18] | Yes | Yes | Yes | Yes | Can’t tell | Can’t tell | Can’t tell | Can’t tell | Yes | Can’t tell | Yes | Yes | Yes | Yes |
| Lee SE,2016[19] | Yes | Yes | Yes | Yes | Can’t tell | Can’t tell | Yes | Yes | Yes | Can’t tell | Yes | Yes | Yes | Yes |
| Song YS,2016[20] | Yes | Yes | Yes | Yes | Can’t tell | Can’t tell | Yes | Yes | Yes | Can’t tell | Yes | Yes | Yes | Yes |
| Sun J, 2016[21] | Yes | Yes | Yes | Yes | Can’t tell | Can’t tell | No | No | Yes | Can’t tell | Yes | Yes | Yes | Yes |
| Gandolfi G, 2015[22] | Yes | Yes | Yes | Yes | Can’t tell | Can’t tell | Yes | Yes | Yes | Can’t tell | Yes | Can’t tell | Yes | Yes |
| Liu X, 2014[23] | Yes | Yes | Yes | Yes | Can’t tell | Can’t tell | Can’t tell | Can’t tell | Yes | Can’t tell | Yes | Yes | Yes | Yes |
| Xing M,2014[24] | Yes | Yes | Yes | Yes | Can’t tell | Can’t tell | Yes | Yes | Yes | Can’t tell | Yes | Can’t tell | Yes | Yes |
| Liu X,2013[25] | Yes | Yes | Yes | Yes | Can’t tell | Can’t tell | Can’t tell | Can’t tell | Yes | Can’t tell | Yes | Yes | Yes | Yes |
| Henderson YC,2009[26] | Yes | Yes | Yes | Yes | Can’t tell | Can’t tell | Can’t tell | Can’t tell | Yes | Can’t tell | Yes | Can’t tell | Yes | Yes |
