# Supplementary material for: The Coexistence of Genetic Mutations in Thyroid Carcinoma Predicts Histopathological Factors Associated With a Poor Prognosis: A Systematic Review and Network Meta-Analysis
Source: Front Oncol. 2020 Nov 3;10:540238. doi: 10.3389/fonc.2020.540238 (PMC7682272; doi:10.3389/fonc.2020.540238)
Supplement: Supplementary Table 3 — Assessment of the quality of evidence using the Grading of Recommendations Assessment. [file Table_3.doc]

**Table S3 Assessment of the quality of evidence using the Grading of Recommendations Assessment**

| **Subjects** | **Number of analyses, by outcome** | **Quality assessment** | | | | | **Overall quality**  **of evidence** |
| --- | --- | --- | --- | --- | --- | --- | --- |
|  |  | **Risk of bias** | **Inconsistency** | **Indirectness** | **Imprecision** | **Other consideration** |  |
| **Patients with thyroid carcinoma** | **Lymph node metastasis()** | Very serious* | Not serious | Serious‡ | Not serious | None | **++--Low***‡ |
|  | **Disease stage ()** | Very serious* | Serious† | Serious‡ | Not serious | None | **+--- Very Low***†‡ |
|  | **Distant metastasis()** | Very serious* | Serious† | Serious‡ | Not serious | None | **+--- Very Low***†‡ |
|  | **Extrathyroidal extension()** | Very serious* | Not serious | Serious‡ | Not serious | None | **++--Low***‡ |
|  | **Tumor recurrence()** | Very serious* | Serious† | Serious‡ | Not serious | None | **+--- Very Low***†‡ |
|  | **Mortality rate()** | Very serious* | Not serious | Serious‡ | Not serious | None | **++--Low***‡ |
|  | **Invasion of the thyroid capsule()** | Very serious* | Not serious | Serious‡ | Not serious | None | **++--Low***‡ |
|  | **Multiplicity()** | Very serious* | Not serious | Serious‡ | Not serious | None | **++--Low***‡ |
| **Patients with papillary thyroid carcinoma** | **Disease stage ()** |  |  |  |  |  |  |
|  | **Lymph node metastasis()** | Very serious* | Not serious | Serious‡ | Not serious | None | **++--Low***‡ |
|  | **Extrathyoidal extension()** | Very serious* | Not serious | Serious‡ | Not serious | None | **++--Low***‡ |
|  | **Distant metastasis()** | Very serious* | Serious† | Serious‡ | Serious^¶^ | None | **+--- Very Low***†‡ |
|  | **Tumor recurrence()** |  |  |  |  |  |  |
|  | **Mortality rate()** |  |  |  |  |  |  |
|  | **Invasion of the thyroid capsule()** |  |  |  |  |  |  |
|  | **Multiplicity()** |  |  |  |  |  |  |

*All studies are case-controlled study. †Poor consistency. ‡Indirect comparisons.¶Fewer studies included
